# Body composition and motor function in children born large for gestational age at term

**DOI:** 10.1038/s41390-024-03211-6

**Published:** 2024-04-20

**Authors:** Yuji Ito, Tadashi Ito, Sho Narahara, Hideshi Sugiura, Yuichiro Sugiyama, Tetsuo Hattori, Hiroyuki Kidokoro, Takeshi Tsuji, Tetsuo Kubota, Jun Natsume, Koji Noritake, Nobuhiko Ochi

**Affiliations:** 1 Department of Pediatrics, Nagoya University Graduate School of Medicine, Aichi, Japan; 2 Department of Pediatrics, Aichi Prefecture Mikawa Aoitori Medical and Rehabilitation Center for Developmental Disabilities, Aichi, Japan; 3 Three-Dimensional Motion Analysis Laboratory, Aichi Prefecture Mikawa Aoitori Medical and Rehabilitation Center for Developmental Disabilities, Aichi, Japan; 4 Department of Physical Therapy, Nagoya University Graduate School of Medicine, Aichi, Japan; 5 Department of Neonatology, Anjo Kosei Hospital, Aichi, Japan; 6 Department of Pediatrics, Japanese Red Cross Aichi Medical Center Nagoya Daiichi Hospital, Aichi, Japan; 7 Department of Developmental Disability Medicine, Nagoya University Graduate School of Medicine, Aichi, Japan; 8 Department of Orthopedic Surgery, Aichi Prefecture Mikawa Aoitori Medical and Rehabilitation Center for Developmental Disabilities, Aichi, Japan

## Abstract

**Background:**

This cross-sectional study compared body composition and motor function between children who were born large for gestational age (LGA) and those born appropriate for gestational age (AGA) and to investigate the association between gait quality and other variables.

**Methods:**

Body composition was determined using a bioelectrical impedance analyzer. Motor functions were assessed using one-leg standing time, timed up-and-go test, five times sit-to-stand test, and three-dimensional gait analysis. We compared the results between two groups. We performed multiple regression analysis to evaluate the association between gait deviation index and variables of LGA, fat mass index, and motor functions (adjusted for age and sex).

**Results:**

Children aged 6–12 years who were born LGA at term (*n* = 23) and those who were born AGA at term (*n* = 147) were enrolled. The LGA group had a higher fat mass index (2.9 vs. 2.2, *p* = 0.006) and lower gait deviation index (91.4 vs. 95.4, *p* = 0.011) than the AGA group. On multiple regression analysis, gait deviation index was associated with being LGA and fat mass index.

**Conclusions:**

In school-aged children who were born LGA, monitoring increased fat mass index and decreased gait deviation index could lessen the risk of metabolic syndrome and reduced gait function.

**Impact:**

- Children aged 6–12 years who were born large for gestational age (LGA) at term showed a higher fat mass index and lower gait deviation index than those who were born appropriate for gestational age at term.
- No significant differences in balance function or muscle strength were observed between groups.
- On multiple regression analysis, gait deviation index was associated with being LGA at birth and fat mass index.
- In school-aged children who were born LGA, monitoring increased fat mass index and decreased gait deviation index could lessen the risk of metabolic syndrome and reduced gait function.

## Introduction

Infants born large for gestational age (LGA) at term are defined as those born at 37–41 weeks’ gestation with a birth weight above the 90th percentile for gestational age.^1^ Previous studies have reported that LGA is associated with a high risk of adverse outcomes, such as birth injuries and hypoglycemia in neonates, hospitalization for respiratory infections in early childhood, and malignancies (including breast cancer) and various psychiatric diseases in childhood and adulthood.^2,3^ LGA infants also have a higher risk of being overweight from early childhood to adolescence, which may lead to metabolic syndrome later in life.^4,5^ A recent population-based study reported that the severity of LGA at term is associated with poor gross and fine motor development during early childhood.^2^ These findings suggest that weight, body mass index, body composition, and motor function are recommended targets to closely monitor in children who were born LGA at term.

In school-aged children, motor functions such as muscle strength and balance function are important for daily physical activities. In addition, gait function is a major indicator of practical motor function, and gait quality based on kinematic data of the pelvis and lower extremities is commonly used to objectively and quantitatively evaluate gait function in children.^6–8^ School-aged children can undergo detailed motor function assessments, and early intervention in this age range is important to improve motor outcomes from adolescence to adulthood. However, few reports have assessed various motor functions and their relationship to body composition using actual measurements in school-aged children who were born LGA at term.

This cross-sectional observational study aimed to clarify the characteristics of body composition and motor function in school-aged children who were born LGA at term and compare them to those of children born appropriate for gestational age (AGA) at term. We also investigated the association between gait quality and other variables. Our initial hypotheses were as follows: (1) children who were born LGA at term have a higher body fat mass, poorer balance function, lower muscle strength, and poorer gait quality than those who were born AGA at term; (2) gait quality is associated with being LGA at birth, body fat mass, balance function, and muscle strength.

## Methods

### Study population

The study participants were native Japanese-speaking children aged 6–12 years attending elementary schools in Okazaki City who were recruited for a medical check-up of motor function between February 2021 and April 2023.

The exclusion criteria were as follows: (1) children who were born preterm (<37 weeks’ gestation) or post term (>41 weeks’ gestation), (2) children who were born small for gestational age (birth weight less than the 10th percentile for gestational age) at term, (3) lacking data on one or more perinatal history items, (4) physical disabilities associated with neurological, musculoskeletal, visual, auditory, or cardiorespiratory abnormalities that affect motor functions, and (5) lacking data on one or more medical check-up items other than perinatal history.

The Research Ethics Board of Aichi Prefecture, Mikawa Aoitori Medical and Rehabilitation Center for Developmental Disabilities approved this study. Written informed consent was obtained from all eligible participants and their parents. The current study was conducted according to the ethical principles outlined in the Declaration of Helsinki.

### Medical interviews and physical examinations

During this medical checkup, the participants first underwent medical interviews and physical examinations by a pediatric neurologist and a pediatric orthopedic surgeon.

### Questionnaires

Questionnaires consisted of perinatal history, lifestyle habits, including exercise and diet, and behavioral traits. The information about perinatal history included gestational age, birth weight, multiple births, multiparity, and parents’ age at birth. The current exercise and eating habits were also assessed. Exercise habits included monthly sports and daily physical activity. As an index of physical activity time, moderate to vigorous physical activity duration per week was assessed using the Japanese version of the Physical Activity Questionnaire in the World Health Organization’s Health Behavior in School-aged Children Survey instrument.^9^ Eating habits included the number of meals and snacks consumed per week. The Japanese version of the Strengths and Difficulties Questionnaire was used to assess behavioral development^10^ because previous studies have suggested that behavioral characteristics had a certain impact on motor functions.^11,12^ The total score ranged from 0 to 40 points, with higher scores indicating more behavioral problems.^10^ The information about perinatal history and the Strengths and Difficulties Questionnaire were completed by the parents. The other questionnaires were completed jointly by the parents and the participants.

### Raven’s Colored Progressive Matrices and Picture Vocabulary Test—Revised

Participants underwent Raven’s Colored Progressive Matrices Test^13^ and the Picture Vocabulary Test—Revised (Nihon Bunka Kagakusha Co., Ltd., Tokyo, Japan). Raven’s Colored Progressive Matrices was used to assess perceptual reasoning ability. The Picture Vocabulary Test—Revised was used to assess word comprehension ability. By combining these two tests, we assessed the intellectual abilities of the participants as background information.

### Body composition measurements

A bioelectrical impedance analyzer is noninvasive, user-friendly, reliable tool to evaluate body composition in school-aged children.^14–17^ Well-trained physical therapists or research assistants measured the total body weight (kg), appendicular skeletal muscle mass (kg), total body fat mass (kg), and body fat percentage (%) using a multifrequency (5, 50, and 250 kHz) bioelectrical impedance analyzer (MC-780A, Tanita, Tokyo, Japan) based on a three-compartment (fat mass, total body water, fat-free dry mass) model.^14,15^ Bioelectrical impedance analysis was performed 2 h after meals, as described in the instruction manual. The participants were instructed to stand on the measuring table with their soles in contact with the anterior and posterior foot electrodes. We also instructed the participants to hold the hand electrodes and maintain contact with the thumbs and palms of both hands. Furthermore, we instructed participants that their arms would be extended and slightly abducted away from their bodies by the end of the measurement. We measured the participants’ height using a stadiometer.

The body mass index was calculated as follows:

- Body mass index = weight/height^2^ (kg/m^2^).

The skeletal muscle mass index was calculated as follows:

- Skeletal muscle mass index = appendicular skeletal muscle mass/height^2^ (kg/m^2^).

The fat mass index was calculated as follows:

- Fat mass index = total body fat mass/height^2^ (kg/m^2^).^18,19^

The Japan Society for the Study of Obesity 2022 guideline for the management of obesity disease established the threshold for abnormal body fat percentage in Japanese children as follows: 25% or higher for males aged 6–12 years, 30% or higher for females aged 6–10 years, and 35% or higher for females aged 11–12 years.^20,21^

### One-leg standing time

Static balance function was assessed using one-leg standing time.^22–25^ The participants were instructed to stand on their dominant leg with their eyes open while keeping their non-dominant leg off the floor without touching the dominant leg. The participants were also instructed to hold their arms alongside their trunks. Using a stopwatch, we recorded the end time in seconds when the non-dominant leg touched the dominant leg, the non-dominant leg touched the floor, or participants maintained their posture for 120 seconds.

### Timed up-and-go test

Dynamic balance function and functional mobility were evaluated using the timed up-and-go test. ^22,26–28^ Before the test, we adjusted the chair height such that the knee flexion during the test was at a 90-degree angle. Participants were instructed to sit on a chair. At the start of the test, participants were instructed to rise from the chair, walk 3 m at a usual speed, turn around, walk back to the chair, and sit down. The total time required for each movement series was recorded in seconds using a stopwatch.

### Five times sit-to-stand test

Functional muscle strength of the lower extremities was assessed using the five times sit-to-stand test.^22,29–31^ Before the test, we adjusted the chair height such that the knee flexion during the test was at a 90-degree angle. The participants were instructed to sit on the chair with their arms crossed over their chests. At the start of the test, the participants were instructed to stand up and sit down as quickly as possible, five times in a row, with their arms crossed over the chest. The total time required for the five repetitions of sitting and standing was recorded in seconds using a stopwatch.

### Three-dimensional gait analysis

Gait quality was assessed using instrumented three-dimensional gait analysis. Three-dimensional gait analysis was performed using eight optical cameras (MX-T 20S; Vicon Motion Systems Ltd., Oxford, UK) at a sampling frequency of 100 Hz. According to Conventional Gait Model 2.3, 24 retro-reflective markers were attached to the participants.^32^ The participants were instructed to walk at their usual speed on a flat surface, and the mean of three measurements was used for the analysis.

Gait kinematic data were recorded and analyzed using Vicon Nexus 2.12 and Vicon Polygon 4.4 (Vicon Motion Systems, Ltd.). Gait quality was evaluated using the gait deviation index. The gait deviation index was calculated from gait kinematics data, which consisted of the sagittal, coronal, and horizontal planes in the pelvis and hip; the sagittal plane in the knee and ankle; and the foot progression angle.^6,7^ According to original American data, the normal mean and standard deviation of the gait deviation index are 100 and 10 points, respectively, and less than 80 points is considered pathological in clinical settings.^6,7^ A change of 0.5 standard deviation in gait deviation index is considered clinically significant.^6,33^ We previously investigated the gait deviation index reference values in 424 Japanese school-aged children. The mean (standard deviation) point of gait deviation index was 94.6 (7.5), and the gait deviation index slightly increases with age.^8^

### Sample size

The desired sample size for the multiple regression analysis was calculated using power analysis. We conducted a power analysis using G*Power (Heinrich Heine University of Düsseldorf, Düsseldorf, Germany) with the following settings: test family = *F* tests, statistical test = linear multiple regression, alpha = 0.05, statistical power = 0.95, *f*^2^ = 0.15 (medium effect size), and number of predictors = 7.^34,35^ Based on these assumptions, the desired sample size was 153 for multiple regression analysis.

### Statistical analyses

Statistical analyses were performed using SPSS Statistics, version 29 (IBM Inc., Armonk, NY). First, we checked for normality using the Shapiro–Wilk test. Second, we compared clinical characteristics, body composition, and motor functions between the LGA and AGA groups. Parametric variables were compared using a two-sample *t* test, and nonparametric variables were compared using the Mann–Whitney *U*-test. Sex, multiple births, and multiparity were compared using the chi-square test or Fisher’s exact test. Finally, we assessed whether the gait deviation index was associated with being LGA at birth (LGA = 1, AGA = 0), fat mass index, one-leg standing time, timed up-and-go test, and the five times sit-to-stand test using forced entry multiple regression analysis. Results were adjusted for age and sex (male = 1, female = 0). The statistical significance level was set at *p* < 0.05 (two-sided).

## Results

**Fig. 1:**
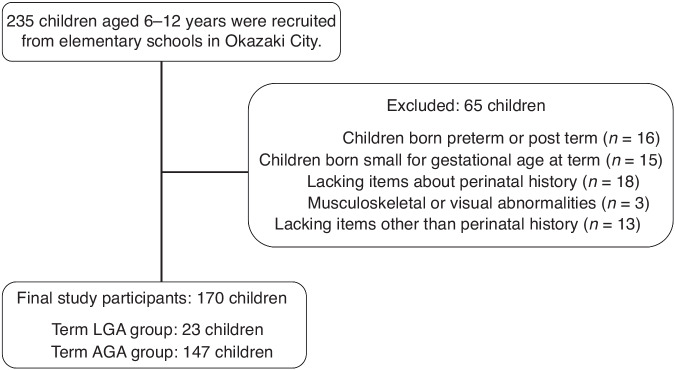
Flowchart of enrollment of study participants. LGA large for gestational age, SGA small for gestational age.

Initially, 235 children were eligible for inclusion. After excluding 65 children, 170 children were finally included in the study, with 23 in the LGA group and 147 in the AGA group. A flowchart of the inclusion and exclusion process is presented in Fig. 1.

The LGA group had a higher birth weight than the AGA group, and there were no other differences between the two groups (Table 1).

**Table 1:** Clinical characteristics of school-aged children who were born large for gestational age at term (term LGA) and appropriate for gestational age at term (term AGA).

Variables	Term LGA (*n* = 23)	Term AGA (*n* = 147)	*p* value
Age (years)^a^	9 (6–12)	9 (6–12)	0.590
Sex, male: female^b^	11:12	74:73	0.823
Gestational age (weeks)^a^	40.1 (37.3–41.3)	39.6 (37.0–41.6)	0.114
Birth weight (kg)^c^	3.67 (0.25)	2.98 (0.28)	<0.001*
Multiple birth^d^	0 (0%)	3 (2%)	0.645
Multiparity^b^	8 (35%)	65 (44%)	0.395
Mother’s age at birth (years)^a^	32 (27–40)	32 (23–42)	0.162
Father’s age at birth (years)^a^	35 (27–42)	33 (24–55)	0.132
Moderate–vigorous physical activity time (hours/week)^a^	4.0 (0.8–11.5)	4.5 (0.0–22.0)	0.791
Sports costs (yen/month)^a^	4000 (0–20,000)	4400 (0–80,000)	0.576
Number of meals (times/week)^a^	21 (21–21)	21 (14–28)	0.447
Number of snacks (times/week)^a^	7 (1–7)	7 (0–7)	0.749
Strengths and Difficulties Questionnaire (points)^a^	8 (0–22)	8 (0–25)	0.938
Raven’s Colored Progressive Matrices (points)^a^	34 (24–36)	32 (20–36)	0.080
Picture Vocabulary Test—Revised (points)^c^	11.5 (2.9)	11.3 (3.2)	0.823

*p < 0.05, statistically significant.

^a^Data were analyzed using the Mann–Whitney *U*-test, and results are presented as median (range).

^b^Data were analyzed using the chi-square test.

^c^Data were analyzed using a two-sample *t* test, and results are presented as means (standard deviation).

^d^Data were analyzed using Fisher’s exact test.

The LGA group had higher body mass index and fat mass index than the AGA group although there was no difference in skeletal muscle mass index (Table 2). Regarding body fat percentage, 2 of 23 (9%) children who were born LGA and four of 147 (3%) children who were born AGA meet the threshold of the Japanese guideline for the management of obesity.

**Table 2:** Body composition of school-aged children who were born large for gestational age at term (term LGA) and appropriate for gestational age at term (term AGA).

Variables	Term LGA (*n* = 23)	Term AGA (*n* = 147)	*p* value
Height (cm)	135 (116–164, 13.6)	133 (106–173, 14.7)	0.406
Weight (kg)	29.5 (21.7–77.9, 13.8)	28.6 (15.2–61.9, 9.3)	0.132
Body mass index	16.5 (14.5–30.2, 3.4)	16.0 (13.2–28.1, 2.3)	0.009*
Skeletal muscle mass index (kg/m^2^)	6.0 (5.0–8.5, 0.9)	5.7 (4.6–8.2, 0.7)	0.146
Body fat percentage (%)	17.8 (8.8–51.0, 8.8)	13.8 (5.4–45.5, 6.4)	0.006*
Fat mass index (kg/m^2^)	2.9 (1.3–15.4, 2.9)	2.2 (0.7–12.8, 1.6)	0.006*

Data were analyzed using the Mann–Whitney *U*-test, and the results are presented as medians (ranges, standard deviation).

*p < 0.05, statistically significant.

The LGA group had a lower gait deviation index than the AGA group (Table 3). One child who was born LGA and no children who were born AGA scored less than 80 points in the gait deviation index. There were no significant differences in balance function or muscle strength of the lower extremities between the two groups.

**Table 3:** Motor function of school-aged children who were born large for gestational age at term (term LGA) and appropriate for gestational age at term (term AGA).

Variables	Term LGA (*n* = 23)	Term AGA (*n* = 147)	*p* value
One-leg standing time (seconds)^a^	120 (8.3–120.0)	120 (3.3–120.0)	0.401
Timed up and go test (seconds)^a^	7.0 (5.1–8.4)	7.0 (4.9–10.8)	0.596
Five times sit-to-stand test (seconds)^a^	6.3 (4.5–8.7)	5.9 (3.8–10.4)	0.187
Gait deviation index (points)^b^	91.4 (6.6)	95.4 (6.9)	0.011*

*p < 0.05, statistically significant.

^a^Data were analyzed using the Mann–Whitney *U*-test, and results are presented as median (range).

^b^Data were analyzed using the two-sample *t* test, and results are presented as the mean (standard deviation).

On multiple regression analysis, there were significant associations between the gait deviation index and the variables of LGA at birth, fat mass index, and age (Table 4). No multicollinearity was found among the independent variables.

**Table 4:** Multiple regression analysis with gait deviation index as the dependent variable in all participants (*n* = 170).

Independent variables	Coefficient (95% confidence interval)	Standardized coefficient	Variance inflation factor	*p* value
Constant	80.93 (72.88 to 88.99)			<0.001*
Large for gestational age at birth	−1.42 (−5.75 to −0.03)	−0.14	1.05	0.048*
Fat mass index (kg/m^2^)	−0.648 (−1.20 to −0.10)	−0.15	1.22	0.021*
One-leg standing time (s)	0.02 (−0.01 to 0.05)	0.09	1.23	0.288
Timed up and go test (s)	0.38 (−0.49 to 1.26)	0.06	1.19	0.390
Five times sit-to-stand test (s)	−0.09 (−0.85 to 0.67)	−0.02	1.10	0.814
Age (years)	1.40 (0.85 to 1.96)	0.44	1.72	<0.001*
Sex	−0.11 (−2.02 to 1.80)	−0.01	1.69	0.907

Data were analyzed using forced entry multiple regression analysis. In this analysis, gait deviation index was the dependent variable, and large for gestational age at birth (large for gestational age = 1, appropriate for gestational age = 0), fat mass index, one-leg standing time, timed up-and-go test, five times sit-to-stand test, age, and sex (male = 1, female = 0) were the independent variables.

*p < 0.05, statistically significant.

Analysis of variance: *p* < 0.001; Durbin–Watson ratio = 1.859; *R*^2^ = 0.259.

## Discussion

We performed a cross-sectional observational study to clarify the characteristics of body composition and motor function in school-aged children who were born LGA at term. We demonstrated that the LGA group showed a higher fat mass index and a poorer gait deviation index than the AGA group although there were no differences in balance function or muscle strength between the two groups. We showed that gait deviation index was associated with being LGA at birth and fat mass index but not balance function or muscle strength. Based on the results of the present study, it may be important to monitor increased fat mass index and decreased gait deviation index to prevent future risk of metabolic syndrome and reduced gait function in children who are born LGA. Early intervention to address increased fat mass index and reduced gait deviation index might decrease the risk of metabolic syndrome and poor gait function in later life. In children who were born LGA, fat mass index and gait deviation index are valuable clinical indicators that can guide nutritional and exercise interventions.

The most important finding of the present study was that school-aged children who were born LGA at term showed lower gait deviation index than those born AGA at term. Furthermore, gait deviation index was negatively associated with being LGA at birth and fat mass index and positively associated with age. In a previous study, preschool-aged children who were born LGA at term showed delayed gross motor development, including gait function.^2^ Gait deviation index reportedly increases slightly with age, even in school-aged children, although school-aged children show an almost adult-like gait pattern.^8^ The gait deviation index was lower in the LGA group than in the AGA group, and this could be because the delayed gait function of the term LGA group did not completely catch up with that of the term AGA group by school age. Several reports describe greater hip and knee flexion in overweight children during the stance phase and greater pelvic and hip rotation during the entire gait cycle, and these kinematic findings were associated with high body fat percentage.^36,37^ These findings suggest that a high fat mass index makes it difficult to move the lower limbs normally during gait; thus, the gait deviation index deteriorates. A sustained high fat mass index in school-aged children may increase the risk of progressive deterioration of gait function.

Based on actual measured values using one-leg standing time, timed up-and-go test, and five times sit-to-stand test, we found that balance function and muscle strength were not worse in school-aged children who were born LGA at term than in those born AGA. In overweight children, decreased balance function and muscle strength, reduced flexibility, and greater fatigue are associated with adiposity.^38,39^ On the other hand, based on a previous study, the degree of decline in balance function and muscle strength, as well as the degree of overweight, are expected to be proportional to the severity of LGA.^2^ Therefore, the lack of children in this study who were born severely LGA (more than 3 standard deviations) at term may have contributed to the lack of significant differences in balance function and muscle strength between the two groups.

The LGA group had a higher body mass index and fat mass index than the AGA group although there was no difference in skeletal muscle mass index. These findings indicate that it is important to focus on quality, not just quantity, in physical growth. In previous reports, LGA infants had a high risk of being overweight and having cardiovascular metabolic problems during childhood and adolescence.^4,5,40,41^ The findings of the current study were consistent with the results of previous reports. Another study in 2–47-month-old infants showed that higher weight in LGA infants is attributable to higher lean mass.^42^ Therefore, between two and six years of age, muscle mass may gradually decrease and fat mass may gradually increase with growth in children who were born LGA. To control fat mass index in school-aged children, infant-specific early interventions, such as promoting breastfeeding, avoiding calorie-dense and high-protein formula, and probiotic supplementation during prolonged antibiotic treatment should be employed.^41^ It is also important to provide sufficient information on appropriate nutritional management methods based on the latest guidelines for family members and the care providers who support them.

In addition to nutritional interventions, appropriate exercise interventions should be considered for decreased gait deviation index in school-aged children who were born LGA. In a previous study on overweight children and adolescents, a 1-h compound exercise program focusing on lower extremity training was provided twice a week for 12 weeks. After the exercise program was completed, improvements in gait kinematics and muscle strength of the lower extremities were observed.^43^ In another study of children with obesity, a 90-minute compound exercise program using the whole body was provided more than three times per week for 13 weeks. Consequently, the exercise group maintained their gait quality compared with the non-exercise group, whose gait quality deteriorated.^44^ Based on the above findings, combined exercise training that ensures a certain level of intensity and frequency may be useful for improving decreased gait deviation index in children who were born LGA at term.

The current study had several strengths and limitations. First, we did not have detailed information about the pregnancy histories of the participants’ mothers or perinatal histories of the participants, although we could analyze several actual measured values of body composition and motor functions including three-dimensional gait analysis. Therefore, we could not evaluate the associations between the variables of present body composition and motor functions, and detailed histories of pregnancy and perinatal periods such as the mother’s body mass index before pregnancy, the absence or presence of gestational diabetes mellitus, cesarean section, and resuscitation procedures at birth. Second, this was a cross-sectional study, and we could compare different variables simultaneously between two groups. However, a longitudinal study is required to evaluate the developmental trajectories of body composition and motor function in children and adolescents born LGA at term. Third, the age range of the participants varied widely, including both prepubertal and pubertal children. Moreover, the sample size of the LGA group may have been insufficient to appropriately control for a variety of influencing factors relevant to body composition and motor function, despite the number of participants satisfying the desired sample size for the multiple regression analysis. Finally, we measured body composition using a multifrequency bioelectrical impedance analyzer based on the three-compartment model. Recently, bioelectrical impedance analysis based on the four-compartment model, which can measure body composition more accurately, is becoming popular, although the three-compartment model has been used for many years, and its usefulness has been established.^18,45^

## Conclusion

School-aged children who were born LGA at term showed an increased fat mass index and decreased gait deviation index compared to those born AGA at term. LGA at birth and fat mass index were significantly associated with gait deviation index. It may be important to support children who were born LGA and their families in nutritional management and exercise interventions to prevent future risk of metabolic syndrome and reduced gait function.

## Data Availability

The datasets generated during and/or analyzed during the current study are available from the corresponding author on reasonable request.
